# Two novel bi‐allelic *KDELR2* missense variants cause osteogenesis imperfecta with neurodevelopmental features

**DOI:** 10.1002/ajmg.a.62221

**Published:** 2021-05-08

**Authors:** Stephanie Efthymiou, Isabella Herman, Fatima Rahman, Najwa Anwar, Reza Maroofian, Janice Yip, Tadahiro Mitani, Daniel G. Calame, Jill V. Hunter, V. Reid Sutton, Elif Yilmaz Gulec, Ruizhi Duan, Jawid M. Fatih, Dana Marafi, Davut Pehlivan, Shalini N. Jhangiani, Richard A. Gibbs, Jennifer E. Posey, SYNAPS Study Group, Shazia Maqbool, James R. Lupski, Henry Houlden

**Affiliations:** ^1^ Department of Neuromuscular Disorders, Queen Square Institute of Neurology University College London London UK; ^2^ Section of Pediatric Neurology and Developmental Neuroscience, Department of Pediatrics Baylor College of Medicine Houston Texas USA; ^3^ Department of Molecular and Human Genetics Baylor College of Medicine Houston Texas USA; ^4^ Texas Children's Hospital Houston Texas USA; ^5^ Development and Behavioural Pediatrics Department Institute of Child Health and The Children Hospital Lahore Pakistan; ^6^ Division of Neuroradiology, Edward B. Singleton Department of Radiology Texas Children's Hospital Houston Texas USA; ^7^ Department of Medical Genetics Health Sciences University, Istanbul Kanuni Sultan Suleyman Research and Training Hospital Istanbul Turkey; ^8^ Department of Pediatrics, Faculty of Medicine Kuwait University Safat Kuwait; ^9^ Human Genetics Center University of Texas Health Science Center at Houston Texas USA; ^10^ Human Genome Sequencing Center Baylor College of Medicine Houston Texas USA; ^11^ Department of Pediatrics Baylor College of Medicine Houston Texas USA

To the Editor:

In a recent article in the *American Journal of Human Genetics*, biallelic pathogenic *KDELR2* variants were described as a novel cause of autosomal recessive (AR) osteogenesis imperfecta (OI) (MIM: #166200) in four families with six affected individuals (van Dijk et al., 2020). The KDELR family of proteins is important in inter‐organelle communication by regulating protein trafficking between the Golgi apparatus and the endoplasmic reticulum (Capitani & Sallese, 2009). *KDELR2*‐related OI results from the inability of HSP47 (heat shock protein 47) to bind KDELR2, leading to failure of HSP47 to dissociate from collagen type 1. HSP47‐bound extracellular collagen cannot form collagen fibers in individuals with pathogenic biallelic *KDELR2* variants (Figure 1; van Dijk et al., 2020). We read the authors' work with great enthusiasm and would like to share clinical and genetic information from two additional unrelated consanguineous families with three affected children with OI with additional phenotypic features, therefore expanding the phenotypic spectrum of *KDELR2*‐related OI.

**FIGURE 1:**
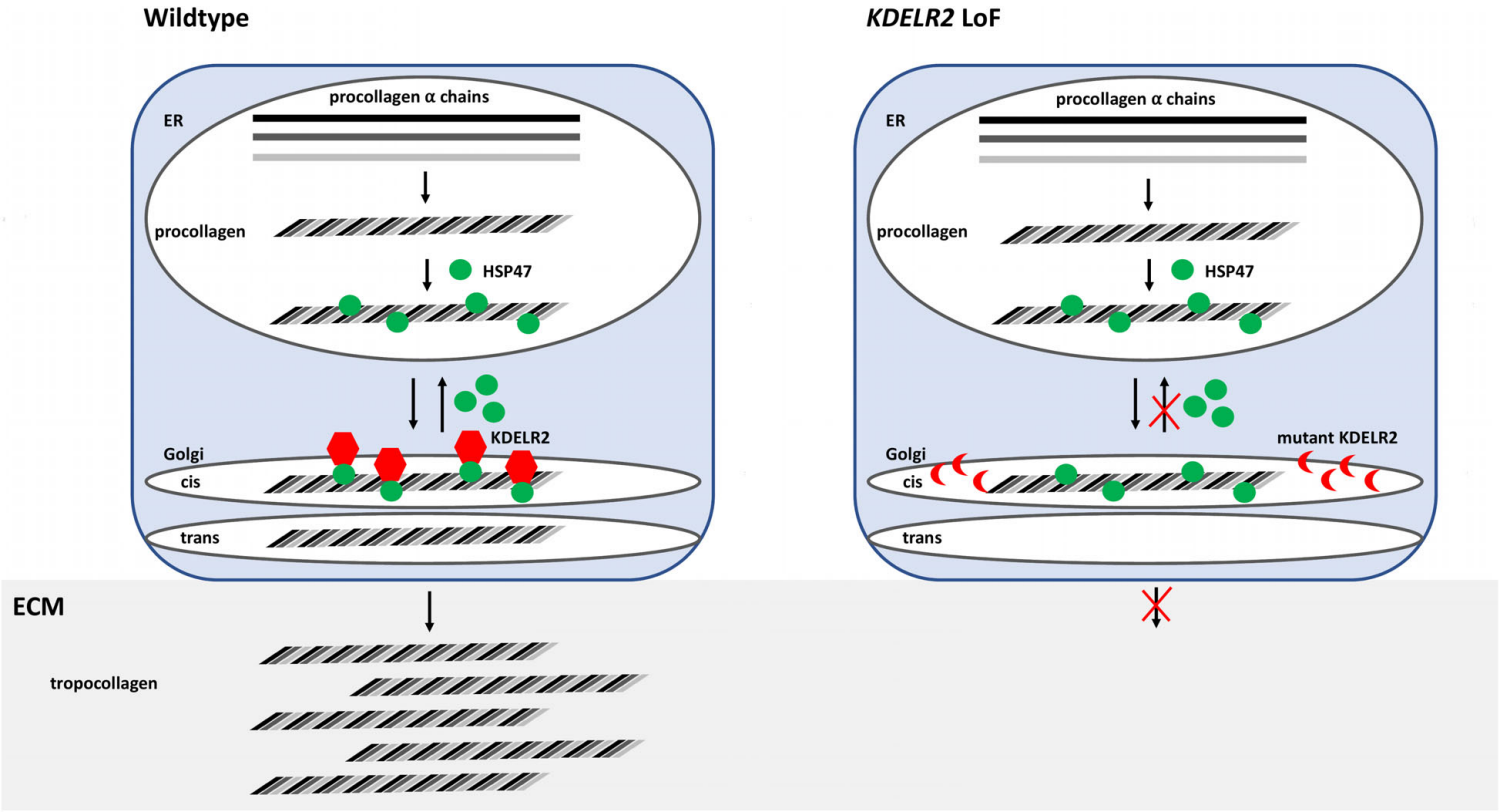
KDELR2 loss of function (LoF) leads to inability of heat shock protein 47 (HSP47) to dissociate from procollagen. In wildtype cells, alpha collagen fibers assemble to form procollagen. Procollagen binds HSP47 and is transferred to the Golgi apparatus where KDELR2 binds HSP47 and leads to dissociation of HSP47 from procollagen. HSP47 is recycled back to the ER. Procollagen is further processed in the Golgi and secreted into the extracellular matrix (ECM) as tropocollagen. In mutant KDELR2 cells, KDELR2 is unable to bind HSP47. HSP47 cannot dissociate from procollagen and is retained in the Golgi and not secreted into the extracellular matrix [Color figure can be viewed at wileyonlinelibrary.com]

OI is a clinically and genetically heterogeneous connective tissue disorder hallmarked by increased susceptibility to bone fractures and is most commonly caused by monoallelic *de novo* pathogenic variants in *COL1A1* (MIM: 120150) or *COL1A2* (MIM: 120160). However, biallelic variants in genes involved in collagen type I biosynthesis have been frequently reported in consanguineous populations (Essawi et al., 2018; van Dijk et al., 2020; Van Dijk & Sillence, 2014). Currently, 20 different types of OI are identified in Online Mendelian Inheritance in Man (OMIM) (Amberger et al., 2015) with variable severity and phenotypic spectrum affecting primarily the skeletal system, although neurodevelopmental and other systemic complications have been observed in some autosomal recessive forms (e.g., *MESD*, MIM: 618644) (Moosa et al., 2019).

Here, we describe three affected children from two unrelated consanguineous families in order to expand the phenotype and further support the role of *KDELR2* in AR OI. Informed consent, including consent to publish photographs, was obtained from the childrens' parents and institutional review board approval was obtained. All three children were clinically diagnosed with progressively deforming OI and neurodevelopmental delay. Three children had motor delay and two of three children had speech delay. The detailed clinical features of each patient are described in Table 1. Pedigrees, radiographs, and brain magnetic resonance images (MRIs) are shown in Figure 2. Common features observed in the affected patients include musculoskeletal abnormalities, including short stature and failure to thrive, Wormian bones, bowed limbs, chest deformity, hypotonia, joint hypermobility, and dysmorphic facies (Figure 2). Family 1 consists of two affected children, a boy and a girl (P1, P2), born to consanguineous (first cousins) parents of Pakistani origin. Both patients have marked motor delay with inability to walk independently at 6 years and 2 years 8 months of age, respectively. The older child crawls as a means of ambulation and has never walked. He has had four fractures in his lifetime, the last at 4 years of age. The younger sister has not had any documented fracture to date at 2 years and 8 months of age. She is not independently ambulatory but can take few steps with great support. In addition, she has speech delay with the first word spoken recently at 2 years of age. Common dysmorphic features in both siblings include epicanthus inversus, deep, sunken eyes, short neck, and thin, sparse hair. Brain MRI obtained from P1 at 6 years of age shows brachycephaly but is otherwise unremarkable (Figure 2(e)). P3 was born to consanguineous first cousin Turkish parents with two prior miscarriages of unknown etiology. He was prenatally suspected to have OI due to ultrasounds showing abnormal bone structure. The patient has one unaffected sibling who does not carry the variant (Figure 2). The patient's first fracture occurred at 21 days of age (Figure 2(d)). Additional features observed include dentinogenesis imperfecta, blue sclera, scoliosis, and neurodevelopmental delay involving both motor and speech. Independent ambulation and speech emerged at 2 years of age; currently at age 4 years he is comparable to his neurotypical peers. Therefore, although he may have had early childhood developmental delay with speech and motor affected, he has caught up to his peers and it is therefore difficult to dissect if the *KDELR2* variant identified contributes to the speech delay observed or if it is due to lack of exposure or other unidentified genetic etiologies. Additionally, at 4 years of age, he is currently independently ambulatory. Neurodevelopmental cognition (developmental quotient/intelligence quotient) of all three patients is unknown nor has formal testing been performed in any of the patients.

**TABLE 1:** Comparison of clinical features in patients with KDELR2‐related osteogenesis imperfecta

	This study			Published in van Dijk et al., 2020					
Individual	P1	P2	P3	P1	P2‐1	P2‐2	P3	P4‐1	P4‐2
Ethinicity	Pakistani	Pakistani	Turkish	Pakistani	Dutch	Dutch	Spanish	Dutch	Dutch
GeneVariant (NM_006854)	c.13C > T (p.Arg5Trp) hmz	c.13C > T (p.Arg5Trp) hmz	c.485A > G (p.Tyr162Cys) hmz	c.448dupC (p.His150fs*24), hmz	c.34C > G (p.His12Asp), hmz	N/A	c.398C > T (p.Pro133Leu), hmz	c.34C > G (p.His12Asp), c.360G > A (p.Trp120*)	c.34C > G (p.His12Asp), c.360G > A (p.Trp120*)
Age, first assessement	4 years 5 months	15 months	24 days	5 years	29 years	N/A	1.5 mo	24 weeks of gestation	N/A
Age, last assessment	6 years	2 years 8 months	4 years 3 months	14 years	39 years	N/A	43 years	N/A	N/A
OFC, first assessment (cm, Z‐score)	47 cm (−2.5)	43 cm (−2.3)	N/A						
Height, last assessment (cm, Z‐score)	77 cm (−3.1)	66.5 cm (−5.2)	83.5 cm (−3)	130 (−4.0)	121 (N/A)	115 (N/A)	138 (N/A)	N/A	N/A
Weight, last assessment (kg, Z‐score)	10 kg (−3.9)	7 kg (−4.1)	10.2 kg (−3.5)	N/A	N/A	N/A	N/A	N/A	N/A
OFC, last assessment (cm, Z‐score)	N/A	N/A	50.5 cm (1.1)	N/A	N/A	N/A	N/A	N/A	N/A
Prenatal fractures	U	U	+	−	−	−	−	+	+
Wormian bones	+	+	+	−	U	U	+	N/A	N/A
Age at first fracture	1 year	N/A	21 days	40	32	U	24	In utero	In utero
Estimated number of sustained fractures	4	0	>2	*N* = 12	*N* = 26	*N* = 15 aged 25 years	*N* > 30	N/A	N/A
Last sustained fracture	4 years 5 months	N/A	4 years	right femur age 10 years	right femur age 28 and right femoral neck age 29	U	right femur, age 37	N/A	N/A
Color of sclera	White	Blue	Blue	White	White	White	White	U	U
Dentinogenesis imperfecta	−	+	+	−	−	−	−	N/A	N/A
Hypermobility of joints	+	+	+	+	+	U	+	N/A	N/A
Hearing impairment	−	−	−	−	−	−	−	N/A	N/A
Chest deformity	Barrel shaped with pectus excavatum	Bell shaped	Barrel shaped, asymmetrical mild carinatum, increased A‐P diameter	Barrel shaped with pectus excavatum	Barrel shaped with pectus excavatum	+	Bell shaped	−	−
Cardiac abnormalities	−	−	mild mitral and tricuspid regurgitation	−	−	+	U	−	−
Vertebral fractures	+	−	+	+	+	U	+	N/A	N/A
Scoliosis	−	+	+	−	+	+	+	−	−
Bowing of upper extremities	+	−	−	−	+	+	+	−	−
Bowing of lower extremities	+	−	+	−	+	+	+	+	+
Shortening of upper extremities	−	−	−	−	+	+	+	+	+
Shortening of lower extremities	+	−	−	−	+	+	+	+	+
Surgical correction for bone deformation	−	−	−	+	+	+	+	N/A	N/A
Age at BP treatment (start/end)	4 years 8 months	N/A	2‐month‐old /still every 6 months	5/9 years	29/37 years	N/A	39/42 years	N/A	N/A
BP type and dosage	Pamidronate 0.5 mg/kg monthly for 8 months	N/A	Pamidronate 0.5 mg/kg every 6 months	Neridronate 2 mg/kg body weight, IV, every 3 months	Alendronic acid 70 mg, weekly	N/A	Zoledronate 5 mg, IV, yearly	N/A	N/A
DEXA scores before BP treatment	N/A	N/A	N/A	Z score: *L2–L4, −3.7; *TBLH, −1.9	Z score: *L2–L4, −3.09; *femoral neck (R), −2.05; *trochanter, −2.50	N/A	U: severe osteoporosis on X‐rays	N/A	N/A
DEXA scores after BP treatment	N/A	N/A	N/A	Z score: *L2–L4, −2.4	Z‐score: *L1–L4, −3.4	N/A	U	N/A	N/A
Calcium—level (mmol/L)	9.9	10.5	9.1	2.36	2.55	U	2.49	N/A	N/A
Alkaline phosphatase at first visit (U/L)	N/A	592	261 (normal for age)	201	69	U	U	N/A	N/A
Alkaline phosphatase at last visit (U/L)	183	368	159 (normal for age)	198	56	U	U	N/A	N/A
Vascular abnormalities	−	−	−	N/A	N/A	N/A	N/A	N/A	N/A
Skin/nail	−	−	−	N/A	N/A	N/A	N/A	N/A	N/A
MRI brain	Brachycephaly, otherwise normal	N/A	N/A, CT head was normal	N/A	N/A	N/A	N/A	N/A	N/A
Mobility	Crawls	Walks with much support	Walks independently	mobile	Wheelchair since age of 4.5 years	Wheelchair	Wheelchair since age of 18 years	N/A	N/A
Intelligence	U	U	U	Normal	Normal	Normal	Normal	N/A	N/A
Hypotonia	+	+	+	N/A	N/A	N/A	N/A	N/A	N/A
Muscle weakness	Mild	Mild	−	N/A	N/A	N/A	N/A	N/A	N/A
Speech delay	−	+	+	N/A	N/A	N/A	N/A	N/A	N/A
Motor delay	+	+	+	N/A	N/A	N/A	N/A	N/A	N/A
Family miscarriages	−	−	2	N/A	N/A	N/A	N/A	N/A	N/A

Abbreviations: hmz, homozygous; U, unknown; N/A, not applicable; BP, bisphosphonate; TBLH, total body less head.

**FIGURE 2:**
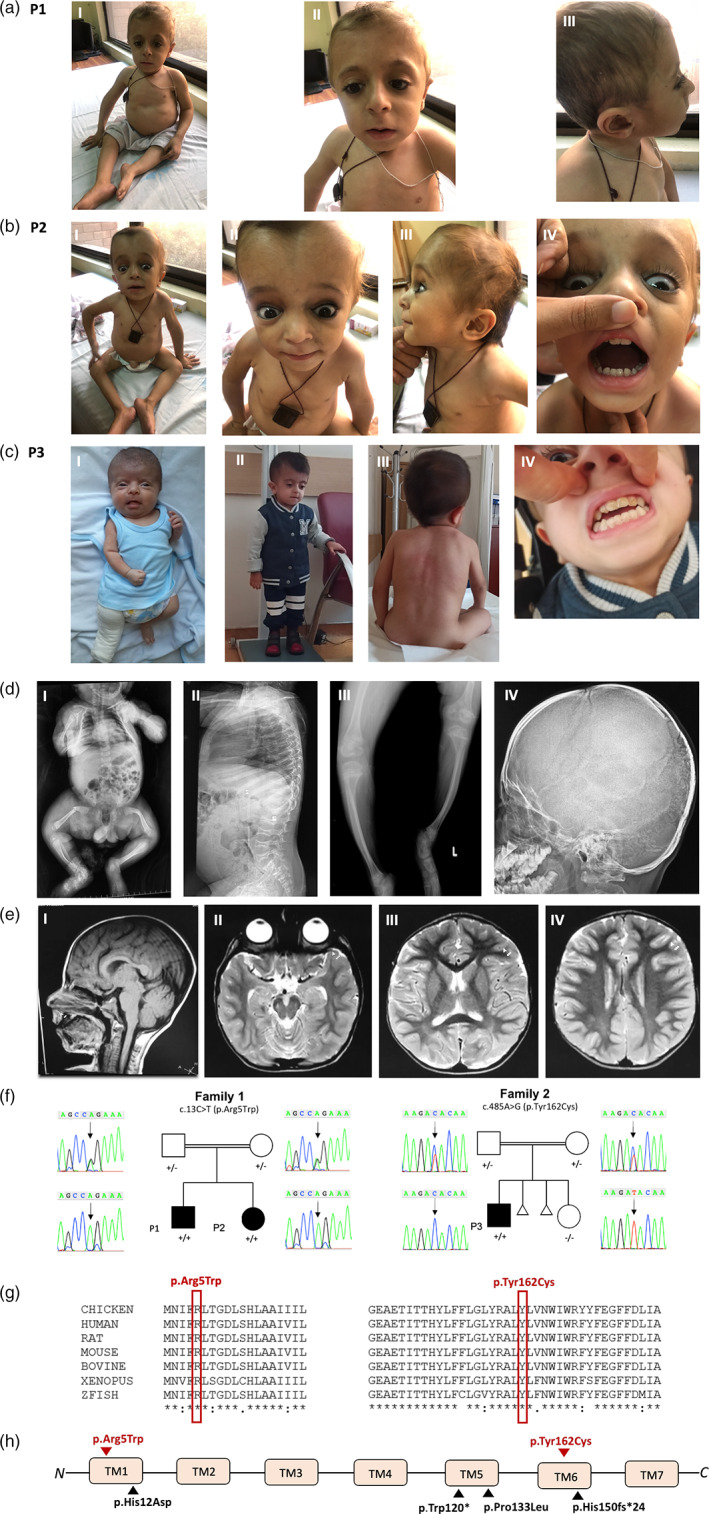
Three affected patients with *KDELR2*‐related osteogenesis imperfecta from two consanguineous families. (a) Photographs of patient P1 showing short stature, barrel shaped chest (I), sunken eyes, epicanthus inversus (II), and sparse thin hair (III). (b) Photographs of P2 showing short stature, barrel shaped chest (I), blue sclera (II), sunken eyes secondary to molding of the soft cranium (II), thin sparse hair (III), and dentinogenesis imperfecta (IV). (c) Photographs of P3 showing infantile short stature a right leg cast following a pathological femoral fracture (I), current short stature at age 4 years (II), scoliosis (III), and dentinogenesis imperfecta (IV). (d) Radiographs of affected subjects depicting infantile femoral fracture from P3 (I), vertebral compression fractures and platyspondyly from patient P1 (II), short bowed limbs from P1 (III), and Wormian bones from P1 (IV). (e) Brain MRI sections from P1 obtained at 6 years of age. (I) Sagittal T1 showing normal brain appearance. (II) Axial T2 showing brachycephaly. (III and IV) Axial T2 images showing age‐appropriate myelination. (f) Sanger segregation of *KDELR2* variants in family 1 and 2. (g) Conservation of amino acid residues across species for both variants. (h) Location of current (red) and previously reported (black) *KDELR2* pathogenic variants. All identified variants to date affect transmembrane domains (TMs) 1, 5, and 6 of the KDELR2 protein product [Color figure can be viewed at wileyonlinelibrary.com]

Family‐based exome sequencing (ES) with rare variant analysis was performed in both families followed by Sanger segregation for the identified variants as described before (Efthymiou et al., 2019; Manole et al., 2020). All three affected subjects were found to have homozygous variants in *KDELR2* (GenBank: NM_006854.3). P1 and P2 have a c.13C > T (p.Arg5Trp) missense variant and P3 has a c.485 A>G (p.Tyr162Cys) missense variant (Table 2). Neither variant is present in gnomAD and both variants are predicted to be pathogenic via in silico prediction analysis (CADD v1.4, MutationTaster, PolyPhen, SIFT). All current and previously reported variants affect highly conserved amino acids located in the KDELR2 transmembrane domains (Figure 1(h)).

**TABLE 2:** Summary of pathogenic *KDELR2* variant alleles

Family	Individual	Ethnicity	Position (hg19)	Nucleotide change	Protein change	Zygosity	gnomAD allele count	REVEL score	CADD score	ACMG classification
This study										
1	P1	Pakistani	Chr7:6523676 G > A	c.13C > T	p.Arg5Trp	hmz	0 htz, 0 hmz	0.64	35	PP1, PM2
1	P2	Pakistani	Chr7:6523676 G > A	c.13C > T	p.Arg5Trp	hmz	0 htz, 0 hmz	0.64	35	PP1, PM2
2	P3	Turkish	Chr7:6505821 T > C	c.485A > G	p.Tyr162Cys	hmz	0 htz, 0 hmz	0.576	32	PM2
van Dijk et al., 2020										
1	P1	Pakistani	Chr7:6505858 G > GG	c.448dupC	p.His150fs*24	hmz	0 htz, 0 hmz	—	—	PM2
2	P2‐1	Dutch	Chr7:6523655 G > C	c.34C > G	p.His12Asp	hmz	0 htz, 0 hmz	0.776	28	PP1, PM2
2	P2‐2	Dutch	Chr7:6523655 G > C	c.34C > G	p.His12Asp	hmz	0 htz, 0 hmz	0.776	28	PP1, PM2
3	P3	Spanish	Chr7:6505908 G > A	c.398C > T	p.Pro133Leu	hmz	0 htz, 0 hmz	0.863	30	PM2
4	P4‐1	Dutch	Chr7:6523655 G > CChr7:6505946 C > T	c.34C > G c.360G > A	p.His12Asp p.Trp120*	cmp htz	0 htz, 0 hmz 0 htz, 0 hmz	0.776; —	2841	PP1, PM2
4	P4‐2	Dutch	Chr7:6523655 G > CChr7:6505946 C > T	c.34C > G c.360G > A	p.His12Asp p.Trp120*	cmp htz	0 htz, 0 hmz 0 htz, 0 hmz	0.776; —	2841	PP1, PM2

Abbreviations: CADD, Combined Annotation‐Dependent Depletion; cmp htz, compound heterozygous; hmz, homozygous; htz, heterozygous; REVEL, rare exome variant ensemble learner.

The role of *KDELR2* in human development has not been well established until this point. However, animal studies of *KDELR2* loss of function (LoF) demonstrate an essential role in embryonic development. The characterization of *Kdelr2*‐LoF mice by the International Mouse Phenotypic Consortium (IMPC)(Dickinson et al., 2016) scored several statistically significant phenotypes, including preweaning lethality, decreased animal size, bone structural abnormalities, abnormalities in head shape and size, facial dysmorphology, and abnormal body wall structure (Table 3), features which overlap with human biallelic pathogenic *KDELR2* variants.

**TABLE 3:** International mouse phenotyping consortium *Kdelr2* LOF phenotypes

**Phenotype**	**Zygosity**	**Life stage**	***p*‐value**
Abnormal embryo size	htz, hmz	E9.5, E18.5	0.00
Abnormal head size	hmz	E18.5	0.00
Abnormal heart looping	htz	E.9.5	0.00
Increased exploratory behavior	htz	early adult	1.17 × 10^−7^
Abnormal bone mineralization	htz	early adult	1.39 × 10^−6^
Abnormal facial morphology	hmz	E18.5	0.00
Preweaning lethality, incomplete penetrance	hmz	early adult	0.00
Abnormal head shape	hmz	E18.5	0.00
Abnormal bone structure	htz	early adult	1.75 × 10^−7^
Abnormal body wall morphology	hmz	E18.5	0.00

Abbreviations: hmz, homozygous; htz, heterozygous.

In conclusion, the data presented here support the role of *KDELR2* in AR OI and expand the phenotypic spectrum of recessive *KDELR2*‐related AR OI first described by van Dijk et al. (2020) to include neurodevelopmental disorders such as motor and speech delay, as well as blue sclerae, dentinogenesis imperfecta, and hypotonia. However, motor delay and hypotonia are common features of OI and one reason they have not previously been reported may have been due to the small sample size of patients with this newly identified genetic etiology of OI. Additionally, it is unclear if the speech delay seen in early development is related to *KDELR2*, lack of exposure, or some other unidentified etiology. Noteworthy, the phenotypic spectrum of IMPC‐generated *Kdelr2*‐LoF mice overlaps with human *KDELR2*‐OI patients and provides a model system in which to better characterize this type of AR OI. Combined data from humans and mouse models could lead to further studies investigating the pathologic mechanism of *KDELR2*‐related OI and to the development of novel disease treatments. With the current rate of novel disease gene discovery and pathogenic disease mechanisms, it is expected that more as of yet undiscovered molecular causes of OI exist. Therefore, it becomes important to perform family‐based genetic analysis in these molecular undiagnosed patients in order to work toward a diagnosis with implications for prognosis, family planning, and potential treatments to mitigate the clinical consequences of this deforming disorder.

## CONFLICT OF INTEREST

The authors declare that they have no conflicts of interest.

## AUTHORS' CONTRIBUTIONS

Stephanie Efthymiou and Isabella Herman performed data collection, analysis, manuscript drafting, and designed the study. Fatima Rahman, Najwa Anwar, Shazia Maqbool, Reza Maroofian, Janice Yip, Tadahiro Mitani, Daniel G. Calame, Jill V. Hunter, V. Reid Sutton, Elif Yilmaz Gulec, Ruizhi Duan, Jawid M. Fatih, Dana Marafi, Davut Pehlivan, Shalini N. Jhangiani, Richard A. Gibbs and Jennifer E. Posey organized participant recruitment and performed data collection. James R. Lupski and Henry Houlden sponsored the research, assisted in study design, and supervised the laboratory studies and clinical integration. All coauthors assisted with manuscript preparation and writing and all coauthors approved of the final manuscript.

## Data Availability

The data that support the findings of this study are available from the corresponding author upon reasonable request.
